# Complete pathological response of NSCLC

**DOI:** 10.1186/1749-8090-8-S1-O227

**Published:** 2013-09-11

**Authors:** K Kovács, C Oláh, G Cserni, R Vizhányó, JP Szekeres

**Affiliations:** 1 Department of Surgery, Bács-Kiskun County Teaching Hospital Kecskemét, Hungary; 2 Depertment of Pathology, Bács-Kiskun County Teaching Hospital Kecskemét, Hungary; 3 Department of Oncology, Bács-Kiskun County Teaching Hospital, Kecskemét, Hungary

## Background

In the last ten years we have operated 35 patients after induction chemotherapy. We would like to present two cases of a pCR of a NSCLC.

## Methods

In a 64y o. male a squamous cell lung cancer was diagnosed in his left upper lobe by bronchoscopic brush biopsy cytology. Stage III.A cT2N2MO. He underwent the neoadjuvant treatment: received six cycles of cisplatin etoposid combination. After the restaging (Stage IBycT2NOMO) we performed a pneumonectomy and the mediastinal blockdissection, because of the involvement of the pulmonary artery and spreading to the lower lobe. The pathologist found only pulmonary fibrosis, but no tumor. In 50 y.o. male an adenocarcinoma of the right upper lobe was diagnosed by bronchoscopic brush cytology. Stage IIIA cT3N2MO. He received the same induction therapy regimen. After restaging (Stage IBycT2NOMO) he underwent a right upper lobectomy. At the operation we have not found any abnormal or enlarged N2 lymphonodes. The hystopathological examination showed only necrotic tissues and haemorrhages, but no tumour. Both patients are alive without any complications and tumourfree.

## Conclusions

As a result of the collaboration with the other pulmonology departments we operate more and more lung cancer patients after neoadjuvant treatment. We can achieve an improvement in the operability and can perform organ-preserving resections as well with the multimodality therapy. In the future we need a special diagnostic method helping in the decision to not perform a resection at all.

